# Cell cycle genes are downregulated after adipogenic triggering in human adipose tissue-derived stem cells by regulation of mRNA abundance

**DOI:** 10.1038/s41598-019-42005-3

**Published:** 2019-04-04

**Authors:** Bruna H. Marcon, Patrícia Shigunov, Lucia Spangenberg, Isabela Tiemy Pereira, Alessandra Melo de Aguiar, Rocío Amorín, Carmen K. Rebelatto, Alejandro Correa, Bruno Dallagiovanna

**Affiliations:** 1 Instituto Carlos Chagas, Fiocruz-Paraná. Rua Professor Algacyr Munhoz Mader, 3775, Curitiba, PR 81350-010 Brazil; 2 Unidad de Bioinformática, Institut Pasteur Montevideo. Mataojo 2020, Montevideo, 11400 Uruguay; 3 Núcleo de Tecnologia Celular, Pontifícia Universidade Católica do Paraná, Rua Imaculada Conceição, 1155, Curitiba, PR 80215-901 Brazil

## Abstract

The adipogenic process is characterized by the expression of adipocyte differentiation markers that lead to changes in cell metabolism and to the accumulation of lipid droplets. Moreover, during early adipogenesis, cells undergo a strong downregulation of translational activity with a decrease in cell size, proliferation and migration. In the present study, we identified that after 24 hours of adipogenic induction, human adipose tissue-derived stem cells (hASCs) undergo a G1-cell cycle arrest consistent with reduced proliferation, and this effect was correlated with a shift in polysome profile with an enrichment of the monosomal fraction and a reduction of the polysomal fraction. Polysome profiling analysis also revealed that this change in the monosomal/polysomal ratio was related to a strong downregulation of cell cycle and proliferation genes, such as cyclins and cyclin-dependent kinases (CDKs). Comparing total and polysome-associated mRNA sequencing, we also observed that this downregulation was mostly due to a reduction of cell cycle and proliferation transcripts via control of total mRNA abundance, rather than by translational control.

## Introduction

Sequential changes in the expression of several genes mark the adipogenic differentiation process and lead to the formation of mature adipocytes^1^. A complex and highly coordinated gene expression program controls adipogenesis triggered by factors that increase cellular cAMP, such as isobutylmethylxanthine (IBMX), insulin, and glucocorticoids in cell culture^2–5^. These specific inducers initiate the transduction of intracellular signals led by the CCAAT/enhancer binding proteins (C/EBPs) and the peroxisome proliferator-activated receptor γ (PPARγ), which are considered master regulators of early adipogenesis^6,7^. The cells can sustain their own differentiation once committed, and the minimum induction time required for the initiation of adipogenesis is three days^8^.

Furthermore different groups showed that cells undergoing adipogenic differentiation have reduced proliferative activity^9–11^. Although this is usually related to contact inhibition, other studies have demonstrated that cell–cell contact is not essential for the occurrence of growth arrest^5^. Moreover, it has been demonstrated that cell cycle genes can regulate genes involved in adipogenic differentiation. The genetic deletion of cyclin D1 lead to a reduction of histone deacetylase (HDAC1)^12^ and promoted the expression of PPARγ and adipogenesis^12,13^. On the other hand, cyclins D3 and G2 were identified as upregulated during adipogenic differentiation, acting as PPARγ coactivators^14,15^.

In our previous work, we used ribosome profiling to better understand the translational regulation involved during the early steps of adipogenesis. We observed that human adipose tissue-derived stem cells (hASCs) treated with adipogenic induction medium for 72 hours had a strong translational regulation. Genes involved in migration, actin cytoskeleton and translational activity were downregulated during early adipogenesis, and this finding correlated with a decrease in cell size, migration and proliferation. Using a methionine incorporation assay, we observed a strong reduction of translational activity that was detected after 24 hours of adipogenic induction^11^. We then hypothesized that the reduction of translational activity previously observed could be related to a decrease in proliferation. Thus, the aim of this work was to analyze the proliferation status and the gene expression profile of the hASCs after 24 hours of adipogenic induction, by assessing both the total and the polysome-associated mRNA. We also investigated the potential role of mTOR inhibitor DEPTOR in this process. The correlation of these data may provide information about the early mechanisms of the adipogenic differentiation process and how it is regulated.

## Results

### Changes in polysome profile and cell cycle arrest occur in hASCs after 24 hours of adipogenic induction

We previously demonstrated that, after 72 hours of adipogenic induction, hASCs strongly downregulate genes related to ribosome biosynthesis and translation. Moreover, using a methionine incorporation assay, we also observed a reduction in translational activity of induced hASCs that could be detected at 12 and 24 hours of treatment with adipogenic medium^11^. Then, to investigate if the reduction of global translational activity was related to the regulation of specific pathways in the first 24 hours of adipogenesis, we used the polysome profiling strategy. We analyzed the fractions of transcripts associated with heavy polysomes and light polysome/monosome, as well as total RNA of hASCs treated with maintenance (non-induced, NI) or induction (I) medium for 24 hours. The multipotency of the hASCs samples used in this study was confirmed by full adipogenic and osteogenic differentiation after 28 days of induction (Fig. S1).

**Figure 1:**
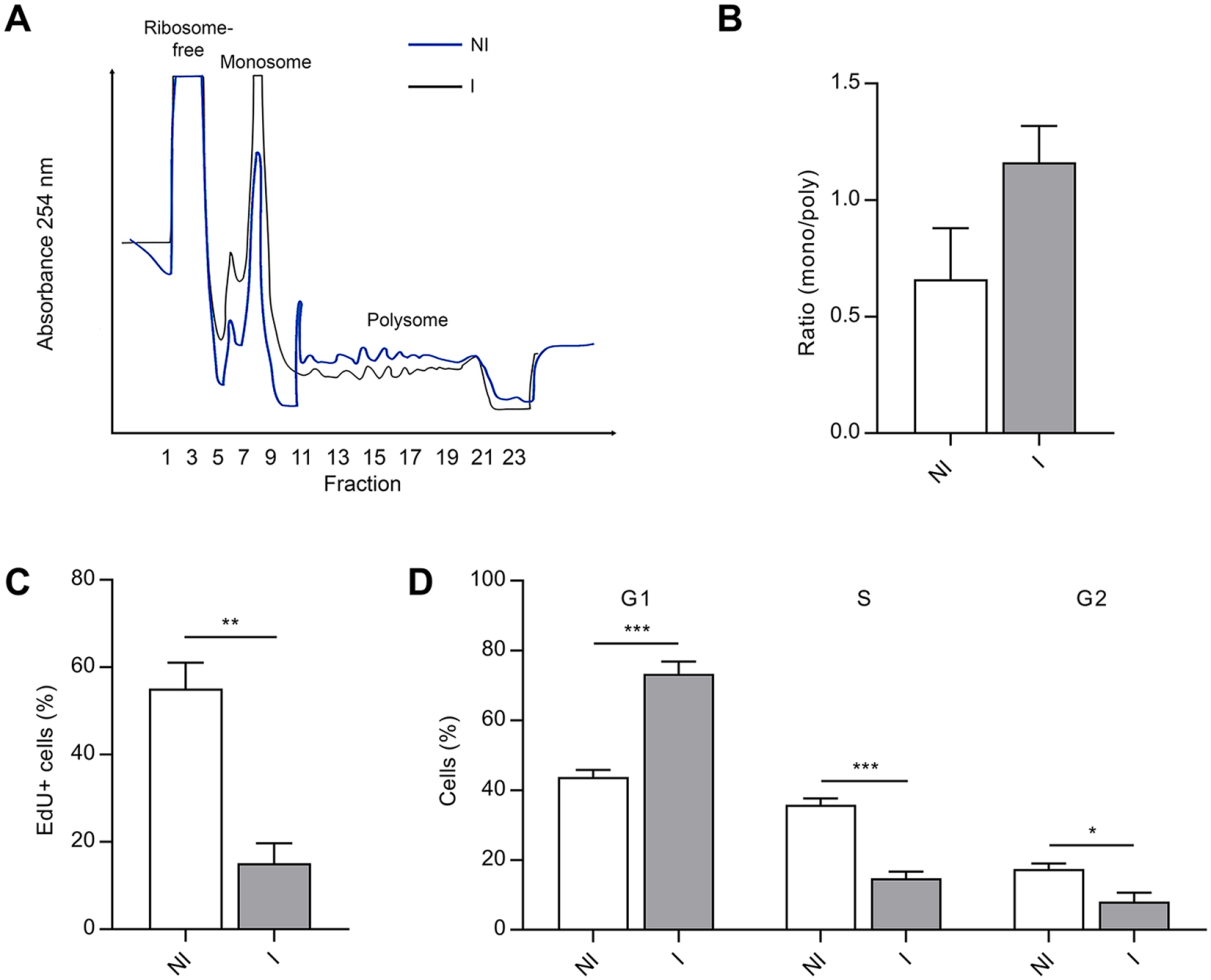
hASCs induced to undergo adipogenesis for 24 hours show a change in the polysome/monosome ratio and have a reduction in proliferative activity. (**A**) The polysome profile obtained by sucrose density gradient of hASCs treated with control (NI, blue line) or adipogenic induction medium (I, black line) for 24 hours. (**B**) Quantification of the ratio between monosomal and polysomal areas (n = 3). Mean with SEM; Shapiro-Wilk normality test: NI p = 0.02; I p = 0.90; Mann-Whitney test. (**C**) Cell proliferation analysis by EdU incorporation assay of hASCs treated with control or adipogenic induction medium for 24 hours (n = 3). Mean with SEM; Student’s unpaired t test analysis: **p < 0.01. (**D**) Cell cycle analysis of hASCs treated with control or adipogenic induction medium for 24 hours. The cells were stained with propidium iodide and analyzed by flow cytometry (n = 4). Mean with SEM; Student’s unpaired t test analysis: *p < 0.05; ***p < 0.001.

Regarding the polysome profile of NI and I hASCs, there was a reduction of the polysomal fraction and an enrichment of the monosomal fraction when adipogenesis was induced (Fig. 1A,B). Previous studies have demonstrated that cells undergoing adipogenic differentiation have reduced proliferative activity^9,10^. To verify if the observed change in the polysome profile correlated with changes in cell proliferative activity in the first 24 hours of adipogenesis, we performed proliferation and cell cycle assays. In all samples, hASCs induced to undergo adipogenesis for 24 hours have a lower proliferative activity than non-induced cells. We found that in the first 24 hours of treatment 55.17% (SEM = ±5.881) of the NI hASCs incorporated EdU while only 15.14% (SEM = ±4.539) of the I hASCs incorporated EdU (Figs 1C and S2A).

To elucidate which phase of the cell cycle was affected, we performed a cell cycle assay. We found that NI hASCs have a mean of 43.78% (SEM = ±2.036) of cells in G1, 35.9% (SEM = ±1.709) in S and 17.48% (SEM = ±1.522) in G2. Alternatively, when adipogenesis was induced, 73.38% (SEM = ±3.490) remained in G1 phase and only 14.73% (SEM = ±1.928) and 8.5% (SEM = ±2.530) continued to S and G2 phases, respectively (Figs 1D and S2B). The results indicate that hASCs undergoing adipogenic differentiation for 24 hours present a change in the polysome profile, with an increase in the monosomal/polysomal ratio, a reduction of the proliferative activity, and an accumulation of cells in G1 phase.

### Decrease in proliferation is DEPTOR-independent

**Figure 2:**
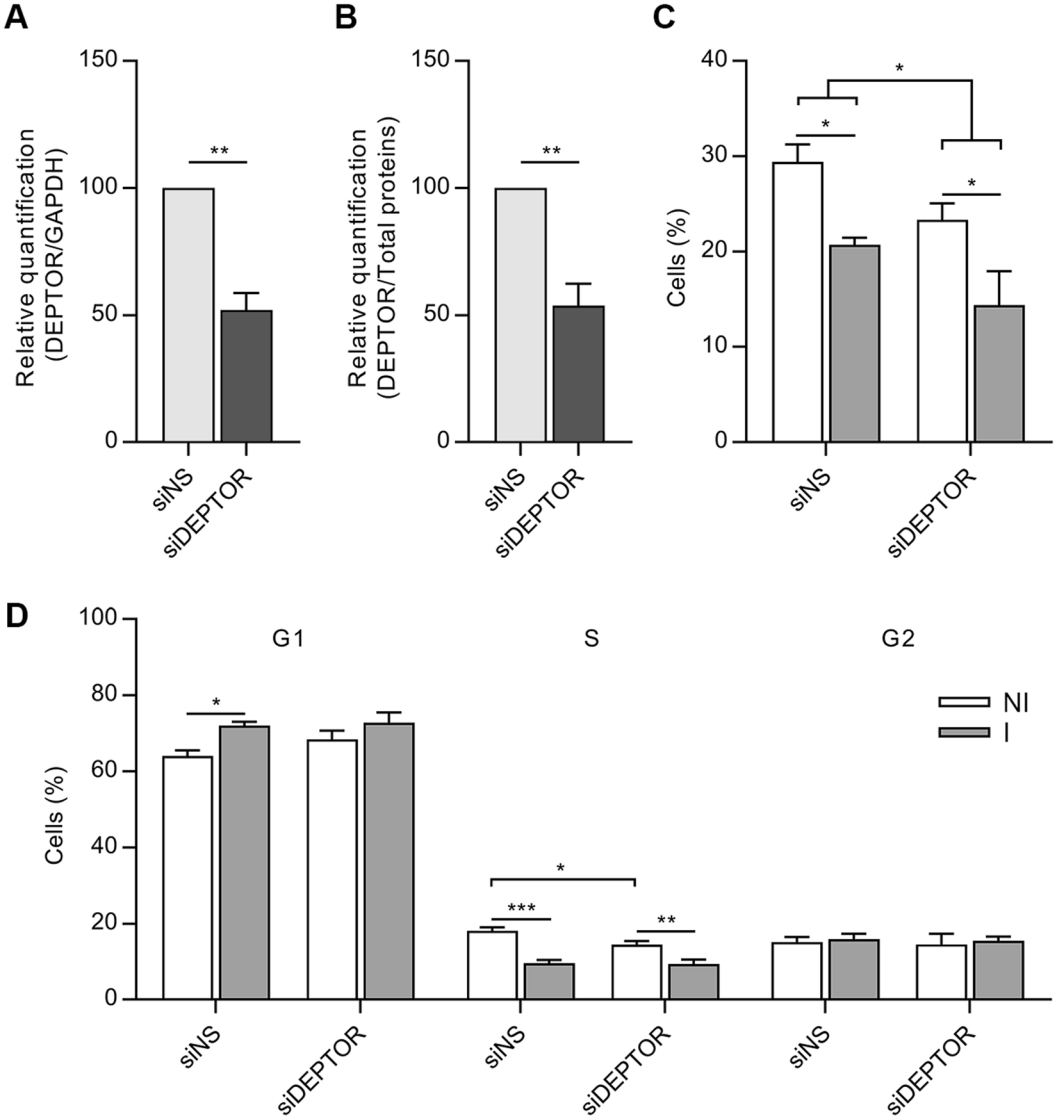
mRNA silencing of DEPTOR in hASCs reduces cell proliferation. (**A**) qRT-PCR of DEPTOR from cells treated with siRNA for DEPTOR or siRNA nonsense (siNS) for 24 hours. The qRT-PCR results were normalized to GAPDH levels (n = 3). Mean with SEM; Student’s unpaired t test analysis: **p < 0.01. (**B**) Quantification of DEPTOR protein expression by Western Blot, results were normalized by total protein (n = 3). Mean with SEM; Student’s unpaired t test analysis: **p < 0.01. (**C**) EdU incorporation assay of hASCs subjected to DEPTOR knockdown for 24 hours and treated with control (NI) or adipogenic induction (I) medium for 24 hours (n = 3). Mean with SEM; Two-way ANOVA, with multiple comparisons: *p < 0.05. (**D**) Cell cycle analysis of hASCs subjected to DEPTOR knockdown for 24 hours and treated with control (NI) or adipogenic induction (I) medium for 24 hours. Cells were stained with propidium iodide and analyzed by flow cytometry (n = 3). Mean with SEM; Two-way ANOVA, with multiple comparisons: *p < 0.05; **p < 0.01; ***p < 0.001.

One of the pathways considered a master regulator of translation and proliferation is mTOR^16–18^. Therefore, we investigated if genes related to the mTOR pathway were differentially expressed in the first 24 hours of adipogenesis. By polysome profiling mRNA sequencing, no significant change was observed in the expression level of mTOR or mTOR pathway-related genes (Table S1), except for DEPTOR, a negative regulator of the mTOR pathway, which was upregulated after adipogenic induction (log2(FC) = 1.58; FDR = 1.64E-08) (Table S1). To investigate if DEPTOR had a role in the cell cycle arrest observed in hASCs during adipogenesis, we used the siRNA methodology. hASCs were transfected with siRNA for 24 hours before adipogenic induction and silencing was confirmed by RT-PCR and Western Blot (Figs 2A,B and S3). At this time point, culture medium was changed to either maintenance or adipogenic culture medium and the cells were incubated for 24 hours. Then, the cells were subjected to cell cycle and proliferation assays. We found that DEPTOR silencing reduced cell proliferation in hASCs treated with either maintenance or induction medium (Figs 2C and S4A). When transfection was performed with scramble siRNAs, 29.43% (SEM = ±1.828) of NI hASCs and 20.73% (SEM = ±0.733) of the ones treated with induction medium were proliferating (n = 3). When silencing with DEPTOR was performed, 23.37% (SEM = ±1.707) of NI hASCs and 14.39% (SEM = ±3.541) of the ones induced for adipogenesis were proliferating (n = 3). Notably, the decrease in proliferation after adipogenic induction was maintained after DEPTOR silencing (Figs 2C and S4A). Alternatively, no significant change was observed in the cell cycle profile after DEPTOR silencing (Figs 2D and S4B). The same pattern was observed for both treatments with maintenance and induction medium (n = 3). However, there was a reduction of cells in S phase when DEPTOR was silenced (maintenance medium). These results suggest that the decrease in cell proliferation of hASCs during early adipogenesis is DEPTOR independent.

### Transcripts related to the cell cycle and proliferation are downregulated after 24 hours of adipogenic induction

**Figure 3:**
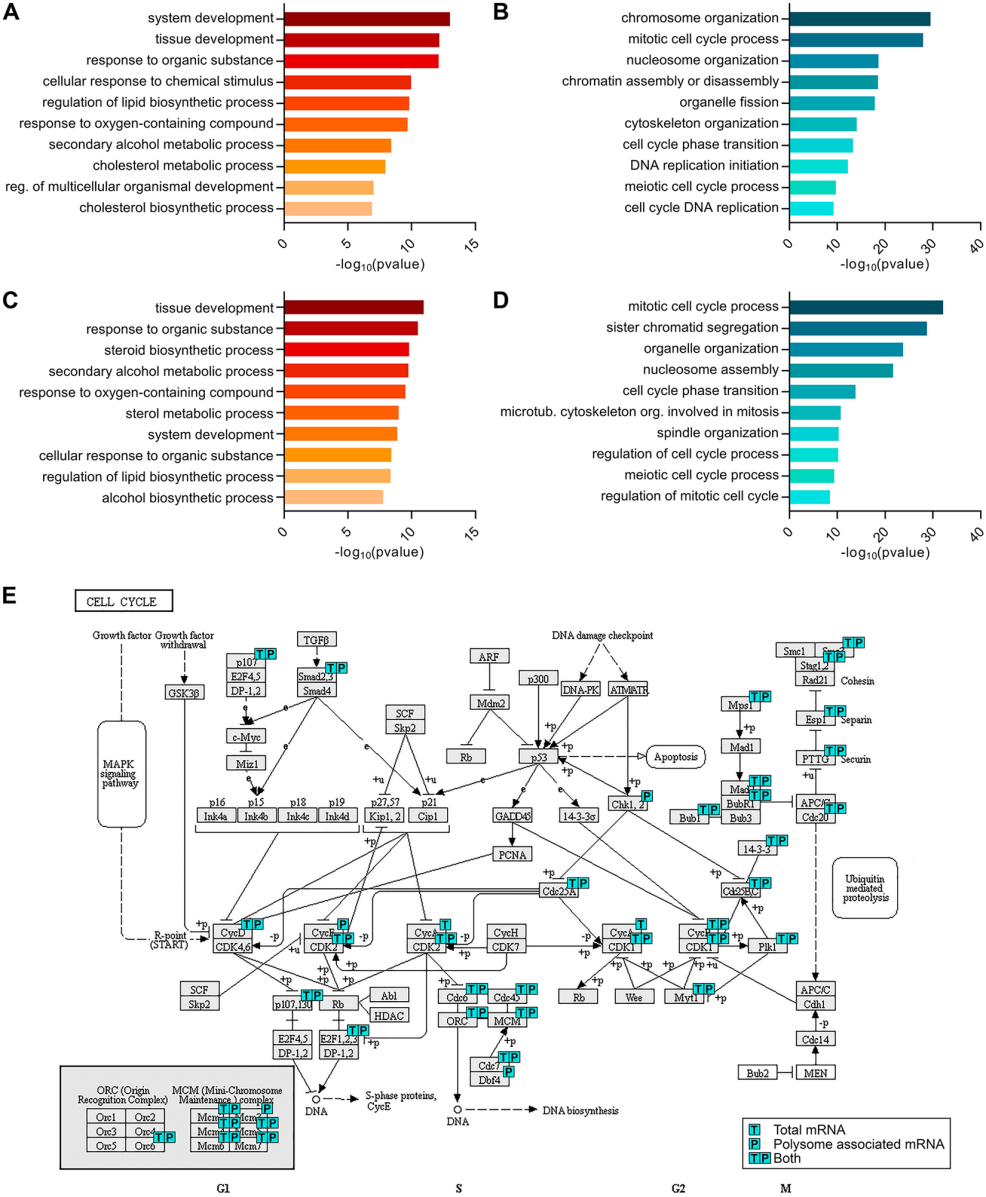
GO analysis of genes up- and downregulated in hASCs after 24 hours of adipogenic induction. (**A**) gProfiler biological process enriched terms for upregulated genes (log(FC) > 1; FDR < 0.01) identified by polysome associated mRNA sequencing. Shown are the 10 terms with lower p values, t depth = 3. (**B**) gProfiler biological process enriched terms for downregulated genes (log(FC) < −1; FDR < 0.01) identified by polysome associated mRNA sequencing. Shown are the 10 terms with lower p values, t depth = 3. (**C**) gProfiler biological process enriched terms for upregulated genes (log(FC) > 1; FDR < 0.01) identified by total mRNA sequencing. Shown are the 10 terms with lower p values, t depth = 3. (**D**) gProfiler biological process enriched terms for downregulated genes (log(FC) < −1; FDR < 0.01) identified by total mRNA sequencing. Shown are the 10 terms with lower p values, t depth = 3. (**E**) Cell cycle related genes identified as downregulated by total and/or polysome associated mRNA sequencing (KEGG).

Further investigating the polysome profiling data, we found that 727 genes were upregulated and 979 were downregulated at the polysomal level during early adipogenesis. Gene ontology (GO) analysis of transcripts identified as up- and downregulated was performed using gProfiler^19^ and DAVID^20,21^. Genes related to response to organic substance and chemical stimulus, system, tissue and multicellular organismal development, and lipid metabolism were upregulated after 24 hours of adipogenic induction (Figs 3A and S5A and Tables S4 and S5). Additionally, the transcripts of adipogenesis-related transcriptional factors C/EBP-β^6,22^ and C/EBP-δ^6^ increased their association with polysomes in the first 24 hours of adipogenesis (Table S1).

Transcripts related to mitosis, chromosome and cytoskeleton organization, DNA replication and the cell cycle were downregulated after 24 hours of adipogenesis (Figs 3B and S5B, and Tables S4 and S5). This notable downregulation of cell cycle-related genes was consistent with the observed reduction of the proliferative activity and cell cycle arrest observed in the first 24 hours of adipogenesis (Fig. 1C,D). The transcripts with reduced expression included cell cycle-related genes, as cyclins B1, B2, D1, E2 and F and the cyclin-dependent kinases (CDKs) 1 and 2. We also observed a downregulation of genes involved in the transcriptional control of cyclin D1, such as E2F-1 and JUN (Table S1). Moreover, we observed a downregulation of genes related to structural changes in the cell during cell cycle, as genes involved in DNA replication, components of DNA polymerase complex, DNA helicases, lamins, histones and kinesins (Table S1).

**Figure 4:**
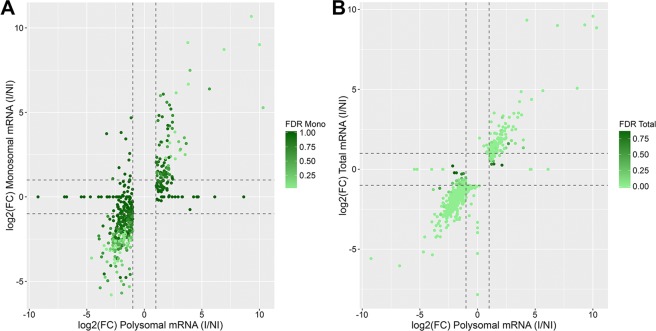
Regulation of transcripts related to the cell cycle and proliferation. (**A**) Cell cycle and proliferation-related genes identified as up- or downregulated in monosome and/or polysome associated mRNA. Scatter plot of log2(FC) in each fraction. (**B**) Cell cycle and proliferation-related genes identified as up- or downregulated in total and/or polysome associated mRNA. Scatter plot of log2(FC) in each fraction.

As the cell cycle arrest was correlated with a change in the polysome profile during early adipogenesis with an increase in the monosome/polysome ratio (Fig. 1A,B), we also analyzed sequencing data of the monosome-associated mRNA. This downregulation of cell cycle genes could be related to a reduced translational rate of these transcripts, with a shift from heavy to light polysomes/monosomes. In monosome associated mRNA, 29 and 14 genes were identified as up- and downregulated after adipogenic induction for 24 hours, respectively (Table S2). By qualitative analysis of differentially expressed genes in monosomal and polysomal fractions, we found that all of the transcripts identified as downregulated in monosomes were also downregulated in polysomes. A similar result was obtained for upregulated transcripts (Fig. S5E). Then, we selected only the genes up- or downregulated (identified by monosomal or polysomal mRNA sequencing) that were included in terms related to proliferation, mitosis or the cell cycle in the GO analysis, which yielded a list of 504 genes differentially expressed. By scatter plot analysis of log2(FC), we found that most genes had a similar regulation in monosome and polysome associated mRNA (Fig. 4A), though with more variability in the monosomal fraction. Additionally, cell cycle arrest was correlated to a strong downregulation of cell cycle and proliferation-associated genes in both heavy and light polysome/monosome fractions.

### Genes related to the cell cycle and proliferation are mainly regulated by control of total mRNA abundance

To verify if the change in the polysome profiling of cell cycle genes was related to mRNA abundance regulation, we analyzed sequencing of total mRNA. After 24 hours of adipogenic induction, 551 genes were identified as upregulated and 942 as downregulated in total mRNA (Table S3). GO analysis revealed that, similar to what was observed in polysomal mRNA, genes related to development and lipid metabolism were upregulated, and genes related to mitosis, chromatid segregation and the cell cycle were downregulated (Figs 3C,D, S5C and S5D and Tables S4 and S5). Additionally, as in the polysome profiling, cyclins A2, B1, B2, D1 and F and CDKs 1 and 2 were downregulated, and cyclins G1 and G2 were upregulated (Table S3) in total mRNA. The decrease in Cyclin D1 mRNA abundance during early adipogenesis of mesenchymal stem cells was previously reported^10,22^. Here we observed that this downregulation correlated with a reduced association of cyclin D1 transcript with polysomes (Table S1) and lead to a decrease in the protein abundance (Fig. S6). Genes involved in transcriptional control of cyclin D1, such as E2F-1 and JUN, were also downregulated, as well as genes related to structural changes during mitosis, such as lamins, kinesins and histones (Table S3). Exploring the KEGG^23–25^ database, cell cycle related genes were downregulated in both total and polysome associated mRNA (Fig. 3E).

Next, we investigated if genes related to cell proliferation and/or the cell cycle were regulated both at the total mRNA and their translational levels (additive regulation) or if the reduced association to polysomes was only reflective of the decrease in the number of transcripts. For this purpose, similar to the analysis performed with the monosomal data, we selected genes up- or downregulated in total or polysomal mRNA that were related to proliferation, mitosis or the cell cycle in the GO analysis, obtaining a total of 584 genes. Using a scatter plot of log2(FC), we found that most genes had a similar regulation in total and polysome associated mRNA (Fig. 4B). This finding suggests that downregulation of cell cycle and proliferation related genes during early adipogenesis was mostly regulated by total mRNA abundance control.

## Discussion

Cell cycle arrest and/or reduced proliferative activity during early adipogenesis have been previously described for 3T3 pre-adipocytes^9^ and mesenchymal stem cells^10^. However, we lack a complete understanding about this entire process and its regulation. Using a 3T3 pre-adipocyte model, different studies have conflicting results on this subject, having found that a prior step of clonal expansion is^26^ and is not^27^ necessary for adipogenic differentiation. Using adipose tissue-derived mesenchymal stem cells as a model, Marquez and collaborators observed that cells undergo a mitotic clonal expansion in the first 48 hours of adipogenic induction^10^. Conversely, in the present work, we observed that reduced proliferative activity could be detected after 24 hours of adipogenic induction and that this reduction was caused by a cell cycle arrest in G1 phase. The differences observed in these studies may be related to the fact that Marquez and collaborators used a period of EdU treatment of 8 hours, while we performed a period of incubation of 24 hours with EdU. Our aim was to access the balance of proliferation after this period and match with the polysome profiling analysis. Therefore, changes in the proliferation status in shorter periods of time may not be noticed in our model.

Working with hASCs induced to undergo adipogenesis for 72 hours, we previously showed that after 3 days of treatment cells have a reduced cell size and migration, proliferation and translational activities. Moreover, a decrease in the protein synthesis rate was observed 24 hours after induction and was correlated to a change in the phosphorylation pattern of translational inhibitor 4EBP1^11^. This finding raised the possibility that the cell cycle arrest observed during early adipogenesis could be related to mTOR pathway inhibition. Using polysomal mRNA sequencing, we identified an upregulation of DEPTOR in cells treated with adipogenic medium for 24 hours. DEPTOR interacts with mTORC1 and mTORC2 and inhibits this signaling pathway^28^. Additionally, DEPTOR positively regulates adipogenesis, as it stimulates adipocyte differentiation, leading to an increase in white adipose tissue (WAT) expansion in mice overexpressing this protein and is also upregulated in WAT of obese humans^29^.

Then, we hypothesized that DEPTOR upregulation could be linked to cell cycle arrest during early adipogenesis and that its knockdown could abrogate this effect. We choose 24 hours to ensure DEPTOR silencing since Zhao and collaborators in 2011 have estimated that the half-life of endogenous DEPTOR was less than 12 hours^30^. However, in our study, silencing DEPTOR 24 hours prior to adipogenic induction showed no significant reduction of cell proliferation, both in NI and I hASCs. This finding suggests that cell proliferation reduction is DEPTOR independent. However, we must consider the fact that DEPTOR knockdown might not be sufficient to affect its inhibitory role on the mTOR pathway or that the reduction of DEPTOR is circumvented by activation of other pathways that regulate mTOR and/or proliferation. Notably, in 2013, Zhang and collaborators have demonstrated that, in multiple myeloma cells, a reduction of 50% in DEPTOR mRNA lead to a similar decrease at the protein level and to changes in the profile of downstream elements of mTOR pathway (as in the phosphorylation of 4EBP1 and Akt), as well as a reduction of cell proliferation^31,32^. A similar result was observed in studies with multiple myeloma^28^ and osteosarcoma^33^ cells, where higher levels of DEPTOR knockdown were obtained. On the other hand, DEPTOR influence on the cell cycle and proliferation varies in different cell types. Kazi and collaborators have demonstrated that DEPTOR knockdown in C2C12 myoblasts augmented phosphorylation of pRb, which stimulated G1-S transition^34^. Although our analysis has suggested that cell cycle arrest during early adipogenesis is independent of DEPTOR upregulation, more studies are necessary to better understand this correlation.

To further understand cell cycle arrest during early adipogenesis, we investigated which other mRNAs were associated with total, polysome and monosome fractions after 24 hours of adipogenic induction. Interestingly, we observed that several genes related to the cell cycle and/or proliferation were downregulated. Regarding the polysome profile of NI and I hASCs, there was a clear shift from heavy to light polysome and monosome fractions. The decrease in global translational activity may lead to a shift of mRNA from heavy polysome fractions to light ones^16^. Then, we hypothesized that downregulation of cell cycle and proliferation genes could be related to a reduction of their translational rate and a shift to light polysomes and monosomes. But the monosomal fraction is not restricted to translationally downregulated transcripts. Using ribosome profiling, Heyer and Moore demonstrated that low expression transcripts or mRNAs containing short ORFs (smaller than 590 nt) being actively translated may be associated to monosomes. On the other hand, they also showed that mRNAs regulated by nonsense mediated decay, or containing uORFs that negatively regulates the canonical ORF may also be enriched in monosomal fraction^35^. To investigate the regulation during early adipogenesis of hASCs, we compared the fold change of proliferation and/or cell cycle associated genes in polysome and monosome associated fractions. Almost all of the genes identified as downregulated in the monosomal fraction were also downregulated in the polysome fraction, with similar fold changes; additionally, genes upregulated in the monosome fraction were also upregulated in the polysome fraction. This result suggests that downregulation of cell cycle associated genes was not mainly related to a reduction of their translational rate. Consistent with this observation, there was a strong downregulation in the total mRNA levels of cell cycle transcripts, suggesting that control of mRNA abundance may be the major regulation point for cell cycle arrest during early adipogenesis.

We also demonstrated that cyclins A2, B1, B2, D1, E2 and F and CDKs 1 and 2 were downregulated while cyclins G1 and G2 were upregulated after 24 hours of adipogenesis. Cyclin D1 is a labile protein that participates in the cell cycle G1-S transition^36^ and its expression may be regulated both at the transcriptional level by the MEK-ERK signaling pathway and at the translational level through the mTOR pathway^37^. Interestingly, we have observed a downregulation of genes involved in the transcriptional control of cyclin D1, such as E2F-1^38^ and JUN^39^. This finding reinforces the idea that cell cycle genes are mainly regulated by control of mRNA abundance, via a DEPTOR/mTOR-independent mechanism, in hASCs during early adipogenesis.

The correlation between cell cycle genes and CT maintenance of stemness and differentiation has been explored by different groups. Recently, Liu and collaborators have demonstrated that G1 cyclins (D1, D2, D3, E1 and E2) are not necessary for embryonic stem cell proliferation, but their downregulation affects pluripotency^40^. Furthermore, the involvement of cyclins in adipogenic differentiation has also been reported. While cyclin D1 was downregulated during early adipogenesis, promoting PPARγ expression and adipogenic differentiation^12,13^; cyclin D3 and cyclin G2 were upregulated, acting as coactivators of PPARγ^14,15^.

Our results showed that adipogenic triggering in hASCs leads to a strong downregulation of cell cycle and proliferation genes. This finding was correlated with a decrease in proliferation and G1-cell cycle arrest, which were DEPTOR independent. We also demonstrated that there is a shift in polysome profile with an increase in the monosome/polysome ratio when adipogenesis is induced. Moreover, our results revealed that cell cycle and proliferation transcripts are primarily regulated by control of mRNA abundance during early adipogenesis in hASCs. We have previously demonstrated that during early osteogenesis, genes related to cell cycle are differentially expressed, but no changes in the proliferation status was observed 24 and 72 hours of osteogenic induction^41^. Thus, the early cell cycle arrest and the reduction of the proliferation seem to be related specifically to adipogenesis at least when comparing with hASCs undergoing osteogenesis. Mesenchymal stem/stromal cells such as hASCs are being extensively used in pre-clinical and clinical studies, understanding details on how these cells proliferate and differentiate are crucial for improving their used and manipulation.

## Methods

### Subjects and cell culture

hASCs were obtained from adipose tissue derived from lipoaspirate samples from donors between 17 and 56 years old (Table 1). Tissue collection and cell isolation were performed after donors and parent/legal guardian (for donors under the age of 18 years old) had given informed consent, in accordance with the guidelines for research involving human subjects and with the approval of the Ethics Committee of Fundação Oswaldo Cruz, Brazil (approval number CAAE: 48374715.8.0000.5248).

**Table 1:** Sex, age, weight, height and BMI of the donors of adipose tissue used in this study.

Donor	Sex	Age (years)	Weight (kg)	Height (m)	BMI (kg/m^2^)
A	Female	27	70.9	1.62	27.0157
B	Female	32	55	1.63	20.70082
C	Male	17	83	1.77	26.49303
D	Female	44	69	1.71	23.597
E	Female	17	64	1.58	25.63692
F	Female	46	57	1.58	22.83288
G	Female	56	80	1.79	24.96801
H	Female	27	53	1.6	20.70313

hASC isolation was performed as previously described^42^. Briefly, 200 mL of adipose tissue were washed with sterile phosphate-buffered saline (PBS) (Gibco Invitrogen), digested with 1 mg/mL type I collagenase (Gibco Invitrogen) (30 min, 37 °C, 5% CO_2_) under constant shaking and filtered through a 100-µm then a 40-µm mesh filter (BD Biosciences). The cell suspension was centrifuged (10 min at 800 × *g*, 8 °C) and treated with hemolysis buffer (ammonium chloride 0.83%, sodium bicarbonate 0.1% and EDTA 0.04%) for 5 minutes to remove erythrocytes. The cells obtained were plated at a density of 1 × 10^5^ cells/cm^2^ with DMEM-F12 (Gibco Invitrogen), 10% fetal bovine serum (FBS), 100 U/mL penicillin and 100 µg/mL streptomycin and kept in a humid incubator at 37 °C and 5% CO_2_ for 24 hours. After, non-adherent cells were removed and the culture medium was changed twice a week. All of the tests and experiments were performed with cell cultures at passages 4 to 6.

To assess the differentiation potential of the isolated hASCs, the cells were submitted to adipogenesis and osteogenesis. The adipogenic differentiation was induced using hMSC Adipogenic Differentiation Medium for 3 days and hMSC Adipogenic Maintenance Medium (hMSC Adipogenic Bullet Kit, Lonza) for 4 days; and this cycle was repeated for a total of 28 days. Then cells were fixed and stained with Nile Red (NR) to identify lipid droplets. For osteogenesis, cells were treated with *hMSC Mesenchymal Stem Cell Osteogenic Differentiation Medium* (Lonza) for 21 days, fixed and stained with OsteoImage™ (Lonza) Mineralization kit, following manufacturer’s instructions.

For 24 hours assays, adipogenic differentiation was performed using hMSC Adipogenic Differentiation Medium (hMSC Adipogenic Bullet Kit, Lonza), in accordance with the manufacturer’s instructions. hMSC Adipogenic Maintenance Medium without insulin was used as a control medium.

### Sucrose density gradient separation and RNA isolation

When hASC cultures reached 70% confluence, treatment with maintenance or induction medium for 24 hours was performed. Next, cells were treated with 0.1 mg/mL cycloheximide (Sigma-Aldrich) (10 minutes, 37 °C), detached with trypsin and washed twice with 0.1 mg/mL cycloheximide in PBS. After centrifugation (700 × g; 5 minutes), the cell pellet was resuspended in polysome lysis buffer (15 mM Tris HCl pH 7.4, 15 mM MgCl_2_, 300 mM NaCl, 100 µg/mL cycloheximide, 1% Triton X-100) and incubated for 10 minutes on ice. The cell lysate was centrifuged at 12000 × *g* for 10 minutes at 4 °C and the supernatant was loaded onto 10% to 50% sucrose gradients (previously prepared with BioComp model 108 Gradient Master). The samples were centrifuged at 150000 × *g* (SW40 rotor, HIMAC CP80WX HITACHI) for 160 minutes at 4 °C and the sucrose gradient fractions were separated using the ISCO gradient fractionation system (ISCO Model 160 Gradient Former Foxy Jr. Fraction Collector), connected to a UV detector. Absorbance at 254 nm was monitored and the polysome profile was recorded. The monosome/polysome ratio was quantified by calculating the area beneath the monosome and polysome peaks using ImageJ software.

RNA from polysomal fractions was isolated using the Direct-zol^TM^ RNA MiniPrep (Zymo Research), following the manufacturer’s instructions. Total RNA from cells in the same conditions (treated with maintenance or induction medium for 24 hours) was also extracted using Direct-zol^TM^ RNA MiniPrep.

### cDNA library preparation and sequencing

For cDNA library preparation, 1 µg of total and polysome-associated RNA were used to perform three independent biological sample replicates. The cDNA libraries were prepared using the TruSeq Stranded Total RNA Sample Preparation kit (Illumina, Inc.) and RNA-seq was performed in an Illumina HiSeq platform using an RNA-seq kit, following the manufacturer’s recommendation (Illumina, Inc.).

### Data analysis

Mapping and counting sequencing data were performed with the Rsubread package^43^. Hierarchical clustering of the samples (log of counts plus one) was performed to evaluate biological variability. Each sample was normalized to one million reads to account for library size.

Differential expression analysis was performed using the Bioconductor R package edgeR^44^. For this analysis, we retained only those genes with at least one count per million in at least three samples.

We considered as upregulated the transcripts with log(FC) > 1 and FDR < 0.01, and as downregulated the transcripts with log(FC) < −1 and FDR < 0.01.

Gene ontology (GO) analysis was performed using gProfiler^19^ and DAVID^20,21^, exploring the KEGG database.

### Cell cycle assay

Cells were detached with trypsin, washed with PBS and centrifuged (700 × g, 5 minutes). The cell pellet was resuspended in an ice-cold solution of 70% ethanol and 30% PBS and incubated for 2 hours at 4 °C for fixation. Then, the cells were washed once with PBS and centrifuged. The cells were resuspended in PBS and an equal volume of 2X staining solution (3.4 mM Tris-HCl pH 7.4; 0.1% NP40; 700 U/L RNase A DNase-free; 10 mM NaCl; 30 µg/mL propidium iodide) was added and incubated for 10 minutes. After centrifugation, the supernatant was discarded, and the cells were resuspended in 200 µL of PBS for cell cytometry analysis. Approximately 10,000 events were acquired with a FACSCanto II flow cytometer (BD Biosciences) and analysis was performed with Flow Jo software version 10.0.8r1.

### EdU incorporation assay

For the proliferation assay, cells were treated with 10 µM EdU (in culture medium) for 24 hours. Then, the cells were detached with trypsin, washed, fixed and stained with the Click-iT® EdU Alexa Fluor® 647 Flow Cytometry Assay Kit (Molecular Probes, Thermo Fisher Scientific), following the manufacturer’s instructions. Approximately 10,000 events were acquired with a FACSCanto II flow cytometer (BD Biosciences), and analysis was performed with Flow Jo software version 10.0.8r1.

### Silencing through RNA interference

To reduce DEPTOR expression in the hASCs, RNA interference silencing was performed using Lipofectamine 2000 Transfection Reagent (Invitrogen™) according to the manufacturer’s instructions. All double-stranded siRNAs were designed and synthesized by Origene (Maryland, USA). The cells were initially plated in 6-well plates at a density of 1 × 10^5^ cells/well in DMEM medium until they reached 80–90% confluence. The cells were transfected with 10 nM siRNA against DEPTOR (siDep - SR312133B) or universal scrambled negative control siRNA (siNC -SR30004). After six hours of transfection, the culture medium was changed to remove the transfectant agent. The cells were harvested 24 h after transfection to calculate the mRNA expression levels.

### RNA extraction and qRT-PCR

Total RNA extraction from the hASCs was conducted according to the RNeasy mini kit (QIAGEN), following the manufacturer’s instructions. Reverse transcription was performed from 1 µg total RNA using the ImProm-II™ Reverse Transcription System (Promega) according to the manufacturer’s instructions. The relative gene expression levels of DEPTOR mRNA were assessed using qPCR with the LightCycler ® 96 Instrument (Roche) and SYBR Select Master Mix (Life Sciences). The program with the cycles were as follows: initiation at 50 °C for 2 min, denaturation at 95 °C for 2 min, followed by 45 cycles of denaturation at 95 °C for 15 sec, annealing at 60 °C for 15 sec and extension at 72 °C for 1 min. The primer sequences for human GAPDH (Forward 5′GGCGATGCTGGCGCTGAGTAC3′ and Reverse 5′TGGTTCACACCCATGACGA3′) results in fragments with 149 base pairs whereas DEPTOR (Forward 5′AATCCAGTCAGAGCAGCGGA3′ and Reverse 5′CCATGGTTTTAGGGCCGTGC 3′) results in fragments with 134 base pairs and recognizes both isoforms (1 and 2). The reactions were run in triplicate, and the generated products were analyzed with the LightCycler ® 96 software (Roche). The data were evaluated as 2-ΔΔCq values (Cq indicates the cycle threshold). The results are expressed as the normalization ratio of the relative quantities of the DEPTOR mRNAs to those of the control (NC siRNA).

### Western Blot

For Western Blot analysis, cells were washed with PBS and scraped after addition of denaturing buffer (160 mM Tris-HCl pH 6.8, 4% SDS, 10% b-mercaptoethanol, 24% glycerol and 0.02% bromophenol blue). Protein extracts were subjected to SDS-PAGE, transferred to nitrocellulose membrane and probed with rabbit anti-cyclin D1 (produced in rabbit, Neomarkers, cat#RB-4091-P1, 1:8000) and anti-DEPTOR/DEPDC6 (D9F5) (produced in rabbit, Cell Signaling, cat#11816 S, 1:1000) antibodies. After incubation with secondary antibody anti-rabbit IgG-peroxidase (produced in goat, Sigma, cat#A6154, 1:2500), membranes were analyzed with Novex® ECL HRP Chemiluminescent kit. The signals obtained were quantified with ImageJ software.

### Statistical analysis

All experiments were performed with hASCs from 3 different donors (biological replicates), except for cell cycle analysis, which was performed with hASCs from 4 different donors. Bar graphs represent mean with standard error mean (SEM). Analyses were performed using GraphPad Prism 5 software. Before running statistical analysis, the distribution was assessed using Shapiro-Wilk Normality test. For experiments with two different conditions and gaussian distribution, Student’s T test was performed. When non gaussian distribution was observed, Mann-Whitney test was used. For experiments with grouped analysis and gaussian distribution, two-way ANOVA with multiple comparisons followed by Sidak post hoc test was performed. Values of p ≤ 0.05 were considered significant.

## Supplementary information

Supplementary Figures

Supplementary Table S1

Supplementary Table S2

Supplementary Table S3

Supplementary Table S4

Supplementary Table S5

## Data Availability

The RNA-seq raw data are deposited in the ArrayExpress repository under the number E-MTAB-6298.
