# Supplementary material for: Cell cycle genes are downregulated after adipogenic triggering in human adipose tissue-derived stem cells by regulation of mRNA abundance
Source: Sci Rep. 2019 Apr 4;9:5611. doi: 10.1038/s41598-019-42005-3 (PMC6449374; doi:10.1038/s41598-019-42005-3)
Supplement: Supplementary file 1 — Supplementary Figures [file 41598_2019_42005_MOESM1_ESM.docx]

**SUPPLEMENTARY FIGURES**

**Cell cycle genes are downregulated after adipogenic triggering in human adipose tissue-derived stem cells by regulation of mRNA abundance**

Bruna H. Marcon^1^, Patrícia Shigunov^1^, Lucía Spangenberg^2^, Isabela Tiemy Pereira^1^, Alessandra Melo de Aguiar^1^, Rocio Amorin^2^, Carmen K. Rebelatto^3^, Alejandro Correa^1*^ and Bruno Dallagiovanna^1*^

^1^ Laboratório de Biologia Básica de Células-tronco, Instituto Carlos Chagas - FIOCRUZ-PR, Curitiba, PR, Brazil

^2^ Bioinformatics Unit, Institut Pasteur de Montevideo, Montevideo, Uruguay

3 Núcleo de Tecnologia Celular, Pontifícia Universidade Católica do Paraná, Curitiba, PR, Brazil

*Correspondence should be addressed to Bruno Dallagiovanna, bruno.dallagiovanna@fiocruz.br; or to Alejandro Correa, alejandro.correa@fiocruz.br.

**Keywords**: hASCs, adipogenesis, cell cycle arrest, proliferation, mRNA regulation

**
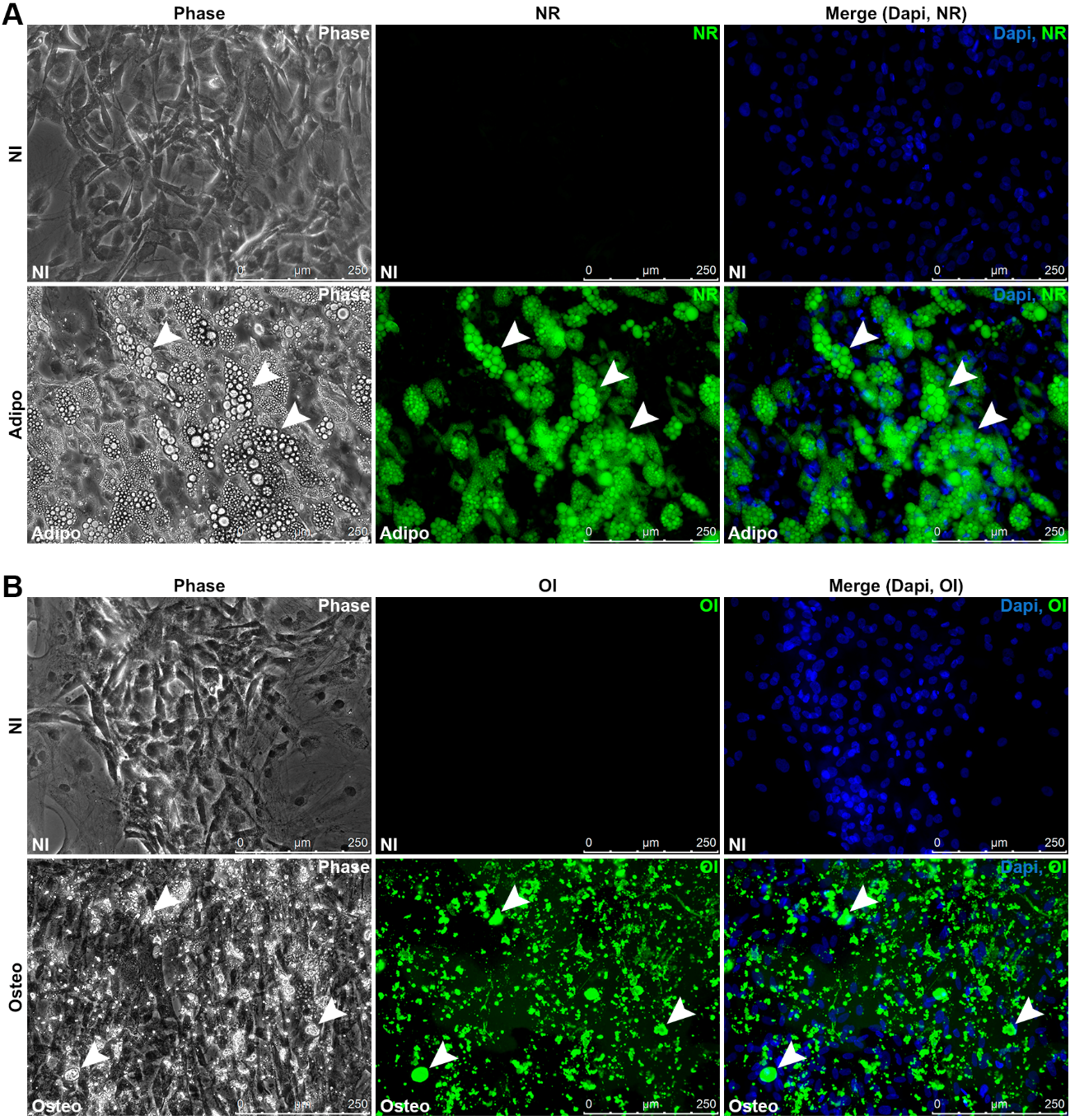
**

**Fig. S1. hASCs obtained from lipoaspirate samples were able to differentiate into mature adipocytes and osteocytes.** (A) hASCs were kept in control medium (non-induced, NI, upper panel) and induced to adipogenesis (Adipo, lower panel) for 28 days. By phase contrast analysis (first column), we could observe that non-induced hASCs have fibroblast-like morphology, while hASCs induced to adipogenesis have lipid droplets (white arrowheads), characteristic of mature adipocytes. Lipid droplets were also stained in green with Nile Red (NR, second column) and nuclei were stained in blue with Dapi (third column shows the merge between Dapi and NR staining). (B) hASCs non-induced (NI, upper panel) and induced to osteogenesis (Osteo, lower panel) for 21 days. By phase contrast analysis (first column), we could also observe that non-induced hASCs have fibroblast-like morphology, while hASCs induced to osteogenesis present hydroxyapatite deposition (white arrowheads). Hydroxyapatite deposition was also stained in green with the kit OsteoImage (OI, second column) and nuclei were stained in blue with Dapi (third column shows the merge between Dapi and OI staining).

**
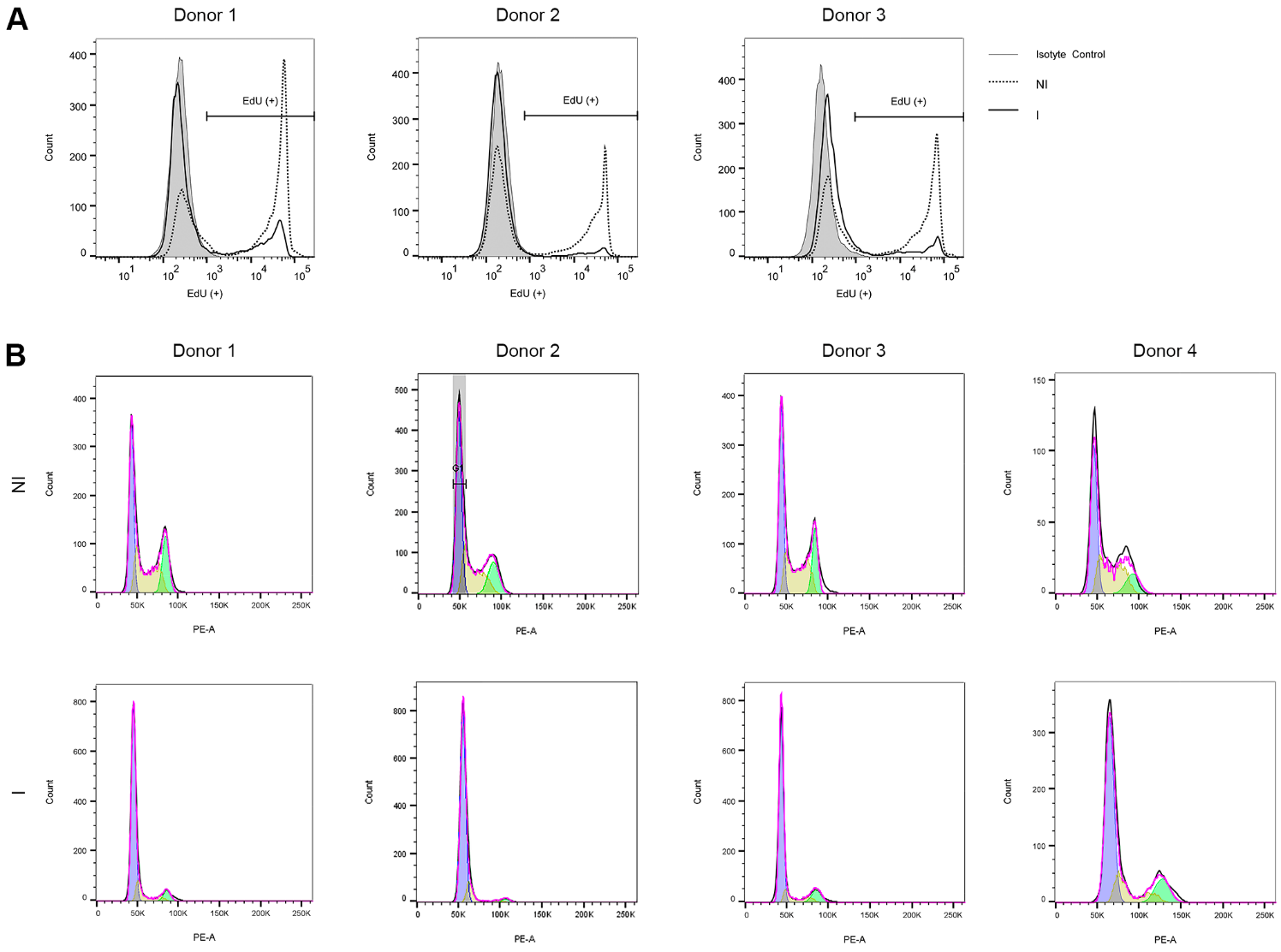
**

**Fig. S2. hASCs induced to undergo adipogenesis for 24 hours have decreased proliferative activity.** (A) Flow cytometry analysis of hASCs from 3 donors treated with control or adipogenic induction medium for 24 hours and subjected to EdU incorporation assay. (B) Cell cycle analysis of hASCs from 4 donors treated with control or adipogenic induction medium for 24 hours. Cells were stained with propidium iodide and analyzed by flow cytometry.


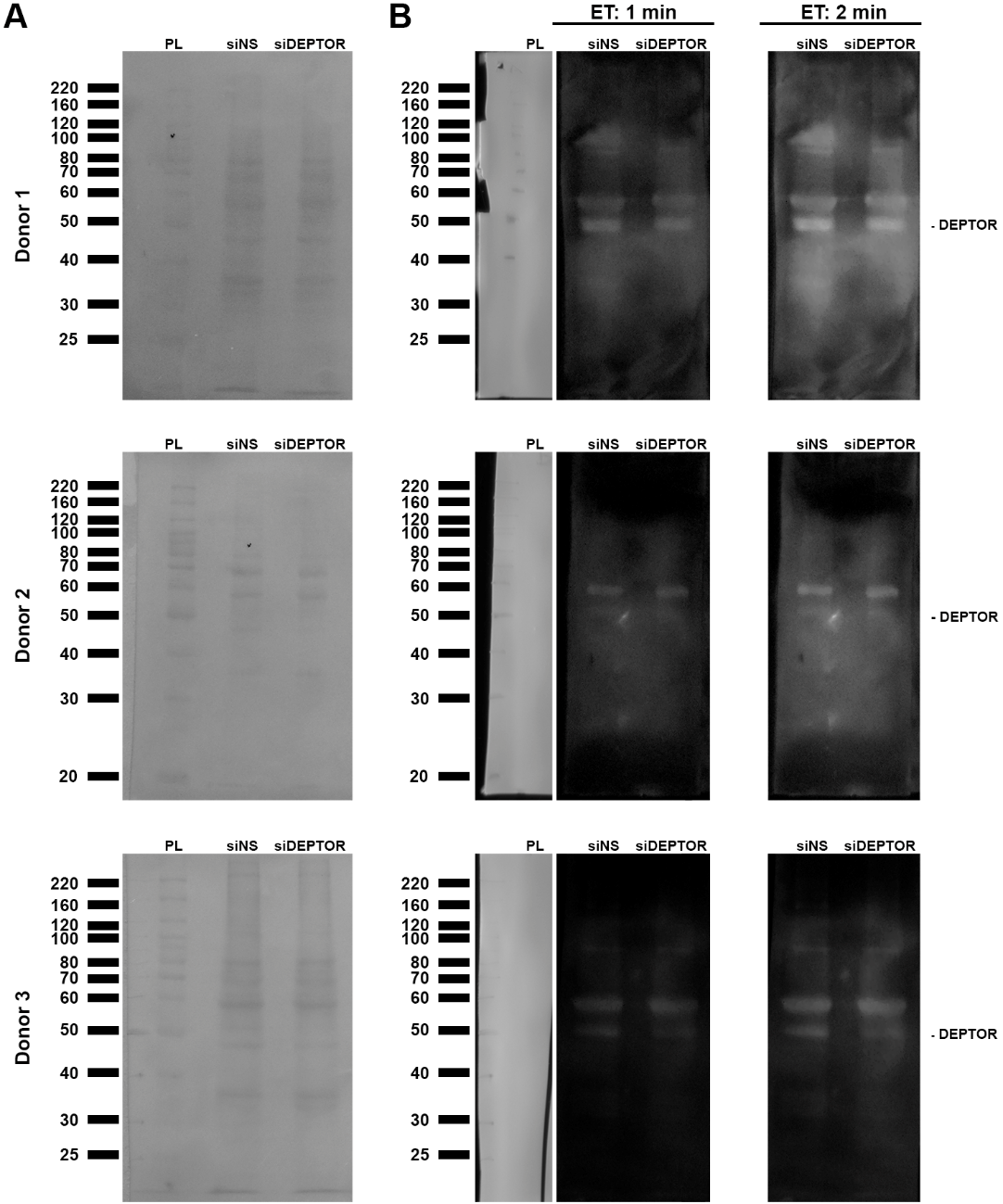


**Fig. S3. DEPTOR protein expression in hASCs after 24 hours of silencing.** (A) Membrane stained with Ponceau showing protein samples from 3 biological replicates of hASCs treated with siRNA for DEPTOR (siDEPTOR) or siRNA nonsense (siNS) for 24 hours. (B) Western Blot for DEPTOR with the same membranes showed in A, with two different exposure times for protein detection. PL= protein ladder; ET= exposure time.


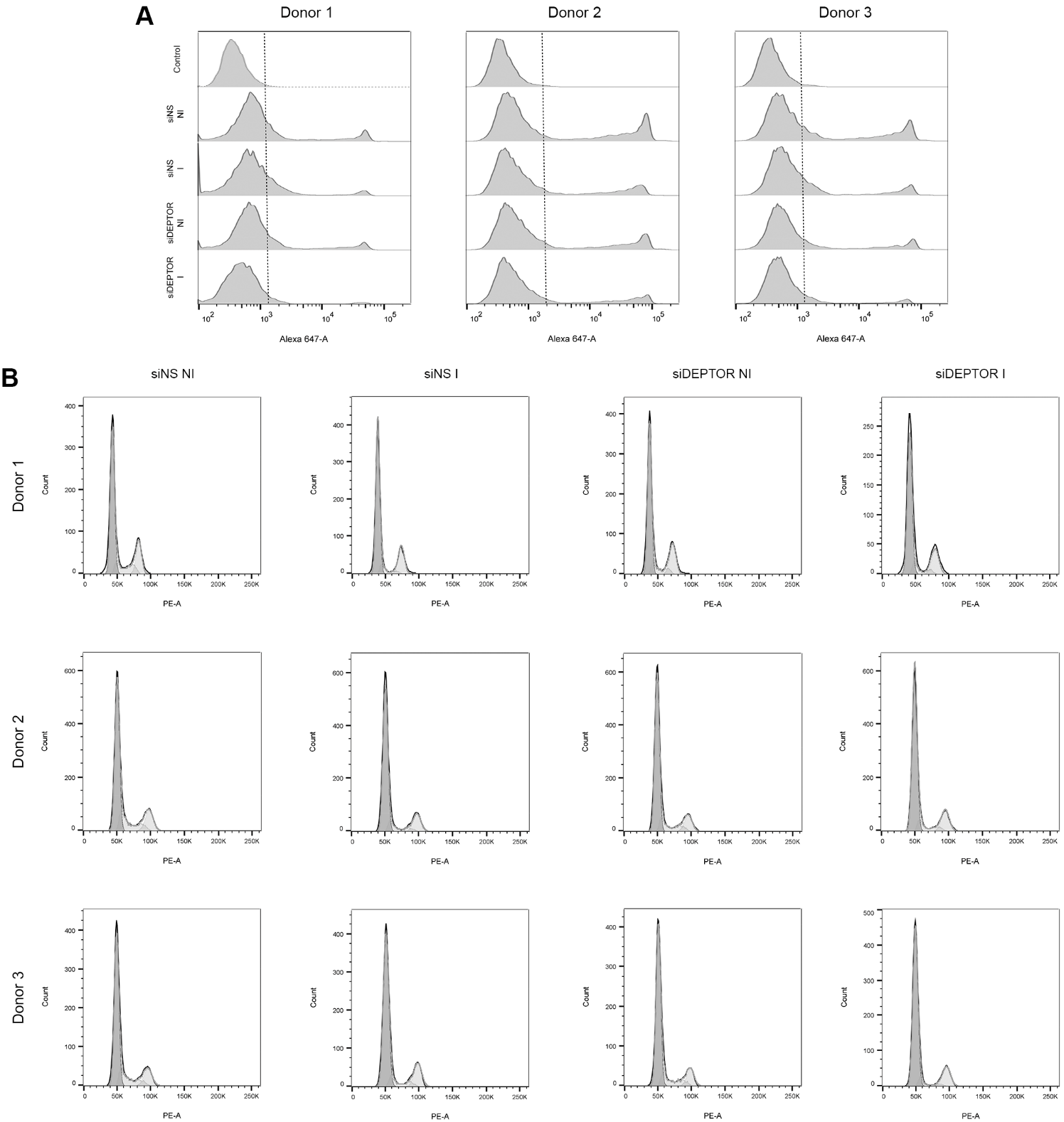


**Fig. S4. mRNA silencing of DEPTOR in hASCs affects the cell cycle and proliferation.** (A) Flow cytometry analysis of hASCs subjected to DEPTOR knockdown for 24 hours and treated with control or adipogenic induction medium for 24 hours and subjected to an EdU incorporation assay (n=3). (B) Cell cycle analysis of hASCs subjected to DEPTOR knockdown for 24 hours and treated with control or adipogenic induction medium for 24 hours. Cells were stained with propidium iodide and analyzed by flow cytometry (n=3).


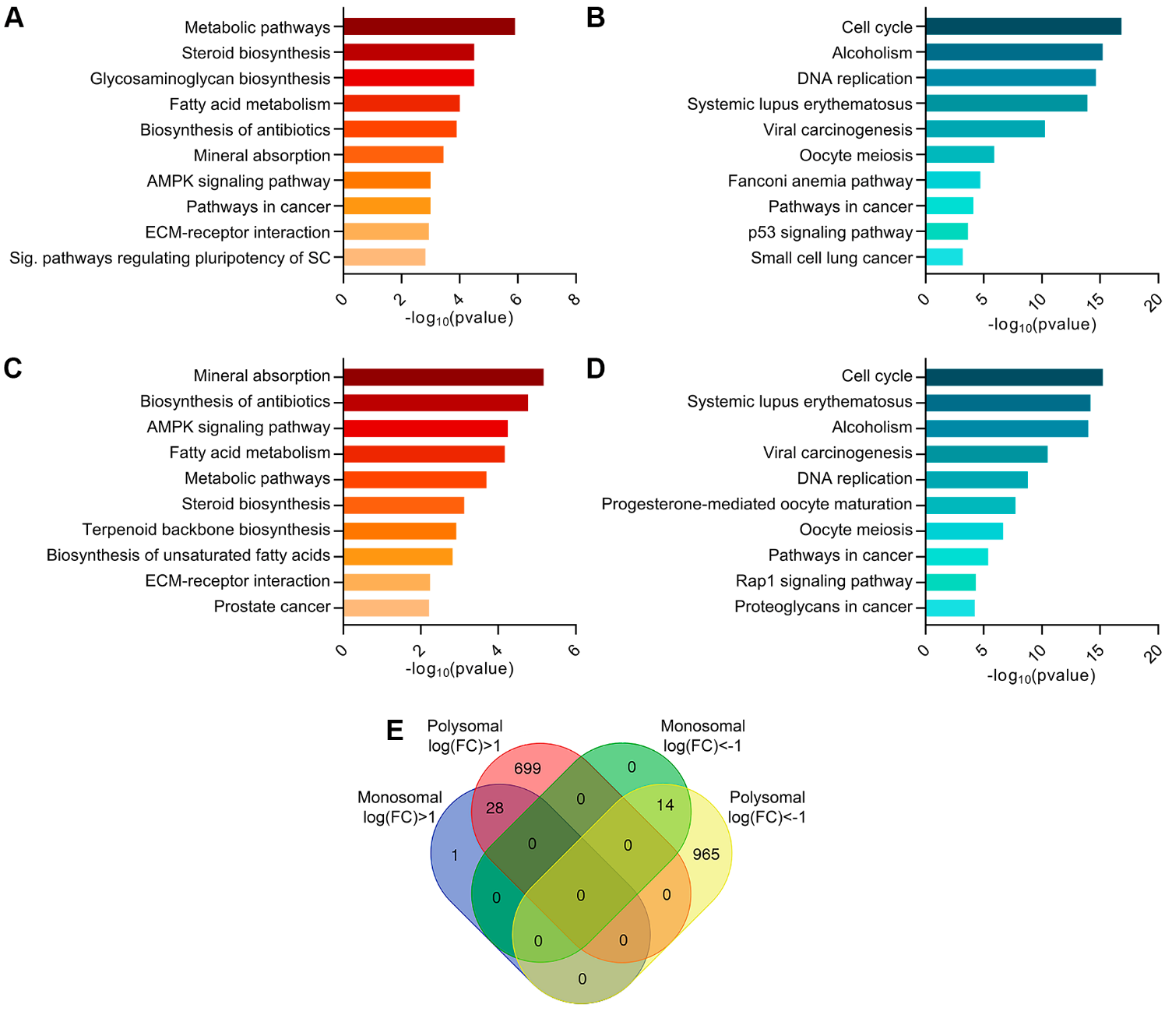


**Fig. S5. GO analysis of genes up- and downregulated in hASCs after 24 hours of adipogenesis.** (A) KEGG enriched terms for upregulated genes (log(FC)>1; FDR<0.01) identified by polysome associated mRNA sequencing. Shown are the 10 terms with lower p values. (B) KEGG enriched terms for downregulated genes (log(FC)<-1; FDR<0.01) identified by polysome associated mRNA sequencing. Shown are the 10 terms with lower p values. (C) KEGG enriched terms for upregulated genes (log(FC)>1; FDR<0.01) identified by total mRNA sequencing. Shown are the 10 terms with lower p values. (D) KEGG enriched terms for downregulated genes (log(FC)<-1; FDR<0.01) identified by total mRNA sequencing. Shown are the 10 terms with lower p values. (E) Venn diagram of genes identified as up- or downregulated in the monosomal or polysomal fractions.

**
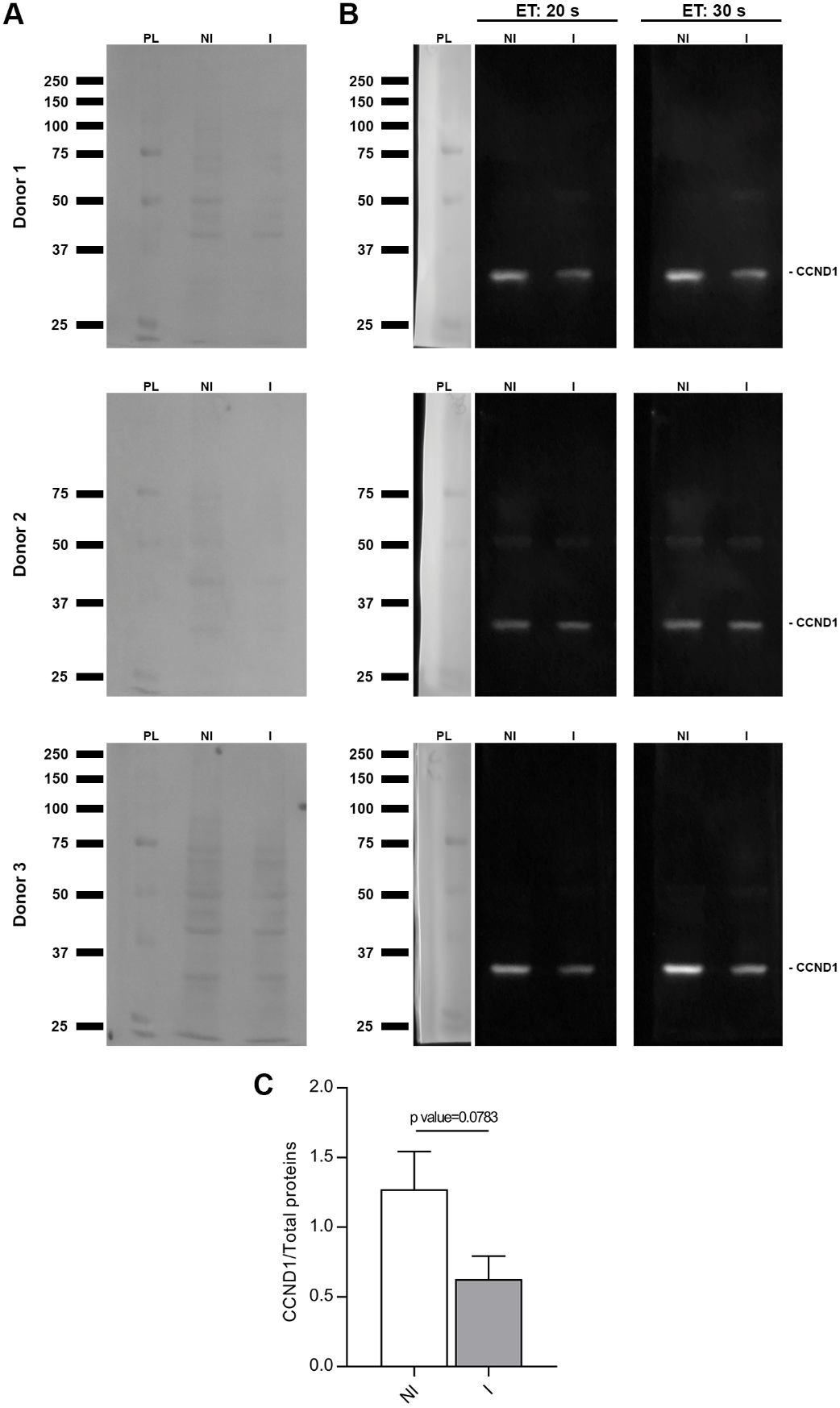
**

**Fig. S6. Cyclin D1 expression in hASCs during early adipogenesis.** (A) Membrane stained with Ponceau showing protein samples from 3 biological replicates of hASCs non-induced (NI) or induced (I) to adipogenesis 24 hours. (B) Western Blot for cyclin D1 (CCND1) with the same membranes showed in A, with two different exposure times for protein detection. PL= protein ladder; ET= exposure time. (C) Quantification of cyclin D1 expression (n=3). Mean with SEM; Student’s paired t test analysis.
